# Patterns of recurrence in adenocarcinoma of the esophagogastric junction: a retrospective study

**DOI:** 10.1186/s12957-020-01917-5

**Published:** 2020-06-27

**Authors:** Haitao Xu, Lianguo Zhang, Jing Miao, Shuai Liu, Hongjian Liu, Teng Jia, Qingguang Zhang

**Affiliations:** 1 Department of Thoracic Surgery, Binzhou Medical University Hospital, No. 661 Huanghe 2nd Road, Binzhou, 256603 Shandong People’s Republic of China; 2 Department of Neonatal Intensive Care Unit, Binzhou People’s Hospital, No. 515 Huanghe 7th Road, Binzhou, 256603 Shandong People’s Republic of China

**Keywords:** Adenocarcinoma of the esophagogastric junction, Recurrence, Surgery, Chemotherapy, prognosis

## Abstract

**Background:**

The prognosis of adenocarcinoma of the esophagogastric junction (AEG) is poor. Understanding the postoperative recurrence pattern of AEG is helpful to verify the effectiveness of treatment and optimize subsequent treatment, so as to improve prognosis.

**Methods:**

This single-center retrospective study included patients with stage III AEG who underwent surgical treatment between January 2009 and December 2016. According to the different postoperative treatment arm, patients were divided into surgery and surgery plus chemotherapy groups. Recurrence-free survival was used as the outcome to compare the recurrence site and pattern between the groups.

**Results:**

In total, were 306 patients enrolled, 123 in the surgery group and 183 in the surgery plus chemotherapy group. During follow-up (median 17.1 months) of 24 months after surgery, 62.0% of patients had tumor recurrence. The overall recurrence rates in the surgery and surgery plus chemotherapy groups were 86.9% and 77.0%, respectively. The recurrence patterns of both groups were mainly distant metastasis. Postoperative chemotherapy reduced the incidence of hematogenous dissemination from 51.2 to 42.0%. Multivariate Cox analysis showed that the pN stage increased the risk of recurrence, while surgery plus chemotherapy reduced the risk.

**Conclusions:**

Patients with AEG have a risk of hematogenous dissemination after surgery. Postoperative treatment arm and pN stage were independent risk factors in patients with AEG. Surgery plus chemotherapy can improve recurrence-free survival and reduce distant metastasis, but they do not have a beneficial role in controlling local recurrence.

## Background

Adenocarcinoma of the esophagogastric junction (AEG) is also known as cardiac cancer in China, and its incidence is increasing in Asian countries including China [1]. Siewert et al. [2] proposed a classification system for AEG. Siewert II and III are the main types in China [3], which differs from the Western countries. Although consensus has been reached in various aspects such as tumor nomenclature, surgical approach, digestive tract reconstruction, and scope of lymph node dissection, there are still many controversies because of its special anatomical location [4, 5].

Surgical resection is the first treatment of choice for AEG. However, clinical trials of AEG are mostly conducted in patients with esophageal cancer [6] or gastric cancer [7, 8], and the recurrence pattern of AEG after surgery is rarely reported. AEG has a poor prognosis [9, 10]. Understanding the recurrence pattern of AEG is helpful to verify the effectiveness of treatment and improve prognosis by optimizing treatment strategies.

Therefore, in this study, we retrospectively analyzed the recurrence sites and patterns of AEG in patients receiving surgery alone and surgery plus chemotherapy, as well as the related factors affecting recurrence-free survival (RFS).

## Methods

### Patients

We retrospectively analyzed patients with AEG who underwent surgery in the Binzhou Medical University Hospital between January 2009 and December 2016. The tumor location was classified according to the Siewert classification system based on the contrast radiography, endoscopy, computed tomography, intraoperative findings, and histological examination. Inclusion criteria were as follows: (1) no residual tumor under microscope; (2) patients receiving surgical treatment alone; (3) patients receiving chemotherapy after surgery, but not radiotherapy; (4) patients receiving no fewer than four cycles of adjuvant chemotherapy after surgery; (5) patients with Siewert type II tumor according to the 7th edition of American Joint Committee on Cancer staging [11], and postoperative pathology stage III; (6) clinical and follow-up data are complete; and (7) no history or coexistence of other malignant tumors. The patients were divided into two groups: surgery group and surgery plus chemotherapy group. This study was approved by the Ethics Committee of the Binzhou Medical University Hospital (No. 2018-WST-2017WS555).

### Surgery and chemotherapy

The choice of surgical approach depended on tumor size, mode of digestive tract reconstruction, experience of surgeons, and general condition of the patients. The surgical approaches included transthoracic partial esophagectomy plus partial gastrectomy and regional lymph node dissection, and transabdominal total gastrectomy plus partial esophagectomy and regional lymph node dissection. Digestive tract reconstruction involved the tubular stomach or jejunum. Patients received the first chemotherapy within 4–6 weeks after surgery. The chemotherapy regimen was intravenous infusion of 40 mg/m^2^ cisplatin for 3 consecutive days and 500 mg/m^2^ fluorouracil for 5 consecutive days for four cycles with an interval of 3–4 weeks [12].

### Tumor recurrence

Recurrence included local recurrence and distant metastasis. Locoregional recurrence was defined as recurrence of primary tumors or in local lymph nodes. Local lymph nodes included lymph nodes around the celiac axis and in the lower mediastinum. Distant metastasis was defined as hematogenous dissemination (including liver, lung, bone, and other parenchymal organs), pleural metastasis, peritoneal metastasis, and distant lymph node metastasis (including nonregional lymph nodes such as neck, axilla, and subclavian). Local recurrence and distant metastasis were judged by chest or abdominal computed tomography, endoscopy, and cytological or histological examination.

### Follow-up

Clinical data were collected through the electronic medical record management system of Binzhou Medical University Hospital. Regular telephone follow-up, combined with outpatient visit data collection was carried out. The last follow-up date was December 30, 2018. The outcome was RFS, which was defined as the time from surgery to recurrence or death. The patient’s age was calculated from the time of operation.

### Statistical analysis

Classified variables were analyzed by the chi-square test and continuous variables were tested by *t* test. The survival curve was drawn by the Kaplan-Meier method. Log rank analysis was used to test the differences in the survival curves. The hazard ratio (HR) and 95% confidence interval (CI) of the survival curves were calculated using the Cox proportional risk model. Univariate and multivariate Cox regression analyses were used to analyze the prognostic factors. The backward-step method was used to optimize the multivariate model. Univariate Cox regression model was also used to analyze the difference in recurrence sites in each treatment group. Statistical analyses were performed using SPSS for Windows version 22.0 (IBM SPSS, Chicago, IL, USA) and STATA for Windows version 15.0 (StataCorp LP, College Station, TX, USA). *P* < 0.05 was considered statistically significant, and all tests were two-sided.

## Results

### Patients

From January 2009 to December 2016, 530 patients with stage III AEG underwent surgery in the Binzhou Medical University Hospital. We enrolled 306 patients, 123 in the surgery group and 183 in the surgery plus chemotherapy group according to the inclusion criteria. Among the 306 analyzed patients, the median age was 63 years. There were 110 men and 13 women in the surgery group, with a median age of 65 years (range 42–79 years). There were 156 men and 27 women in the surgery plus chemotherapy group, with a median age of 62 years (28–81 years). There were differences in age distribution between the two groups. There was no difference in the baseline distribution of gender, tumor size, cT stage, cN stage, pT stage, pN stage, pTNM stage, histological type, and number of resected lymph nodes between the two groups (Table 1).

**Table 1:** Baseline patients characteristics

Characteristics	S (*n* = 123), *n* (%)	S + CT (*n* = 183), *n* (%)	*P* value
Age, years			< 0.001
Median	65	62	
Range	42–79	28–81	
Sex			0.287
Male	110 (89.4)	156 (85.2)	
Female	13 (10.6)	27 (14.8)	
Tumor length, cm			0.084
Median	5	5.5	
Range	1.8–10.0	1.6–12.0	
pT stage/cT stage			0.162
T2	3 (2.4)	3 (1.6)	
T3	82 (66.7)	104 (56.8)	
T4a	38 (30.9)	76 (41.5)	
pN stage			0.932
N0	12 (9.8)	16 (8.7)	
N1	23 (18.7)	38 (20.8)	
N2	39 (31.7)	61 (33.3)	
N3	49 (39.8)	68 (37.2)	
pTNM stage			0.617
IIIa	32 (26.0)	44 (24.0)	
IIIb	32 (26.0)	41 (22.4)	
IIIc	59 (48.0)	98 (53.6)	
Histology			0.770
Well-differentiated AC	6 (4.9)	10 (5.4)	
Moderately differentiated AC	35 (28.5)	53 (29.0)	
Poorly differentiated AC	57 (46.3)	75 (41.0)	
Mucinous AC	25 (20.3)	45 (24.6)	
Method of resection			0.053
TTE	70 (56.9)	124 (66.8)	
TAE	53 (43.1)	59 (32.2)	
Number of resected lymph nodes			0.278
(mean ± SD)	19.21 ± 8.734	20.25 ± 7.848	
cN stage			0.917
N0	18 (14.6)	26 (14.2)	
N1	105 (85.4)	157 (85.8)	

### Patterns of recurrence

**Fig. 1:**
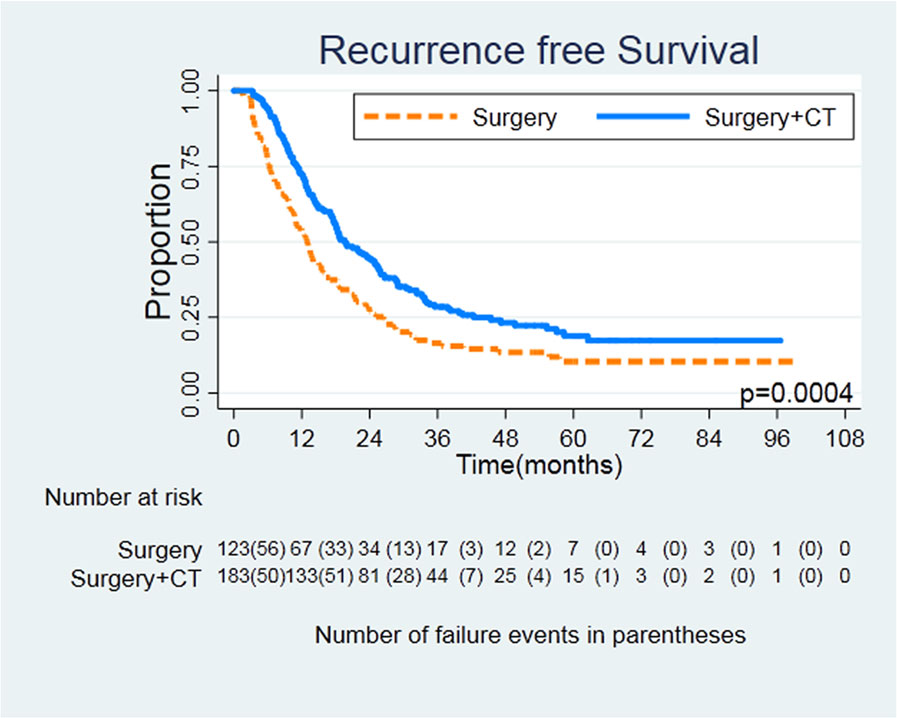
Recurrence-free survival for patients undergoing surgery or surgery + CT. Kaplan-Meier survival curves, HR = 0.637, 95% CI = 0.495–0.820, log rank *P* = 0.0004

**Fig. 2:**
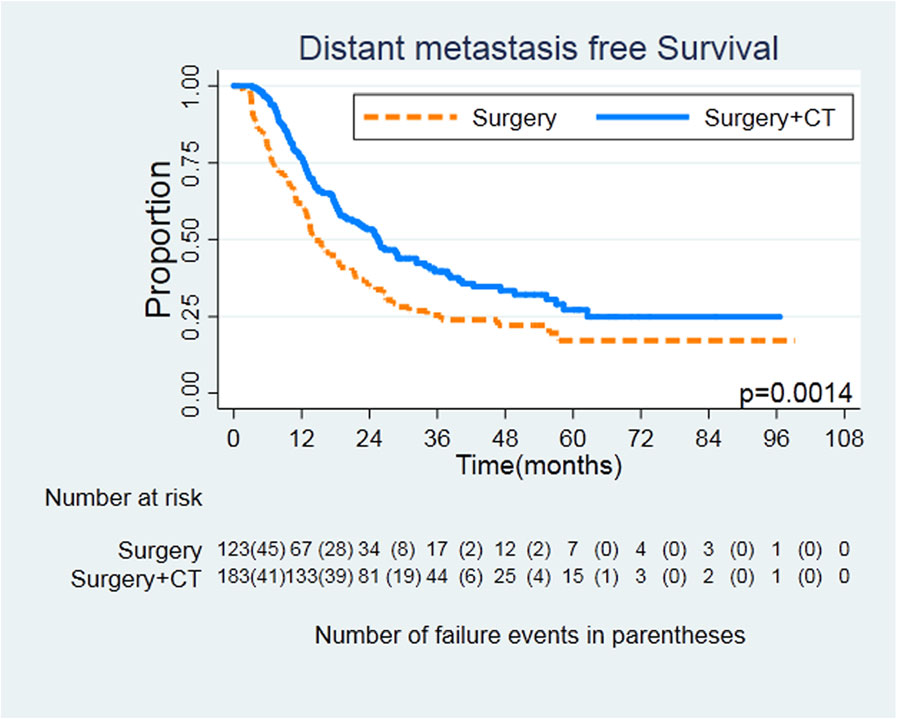
Distant metastasis-free survival for patients undergoing surgery or surgery + CT. Kaplan-Meier survival curves, HR = 0.632, 95% CI = 0.476–0.839, log rank *P* = 0.0014

**Fig. 3:**
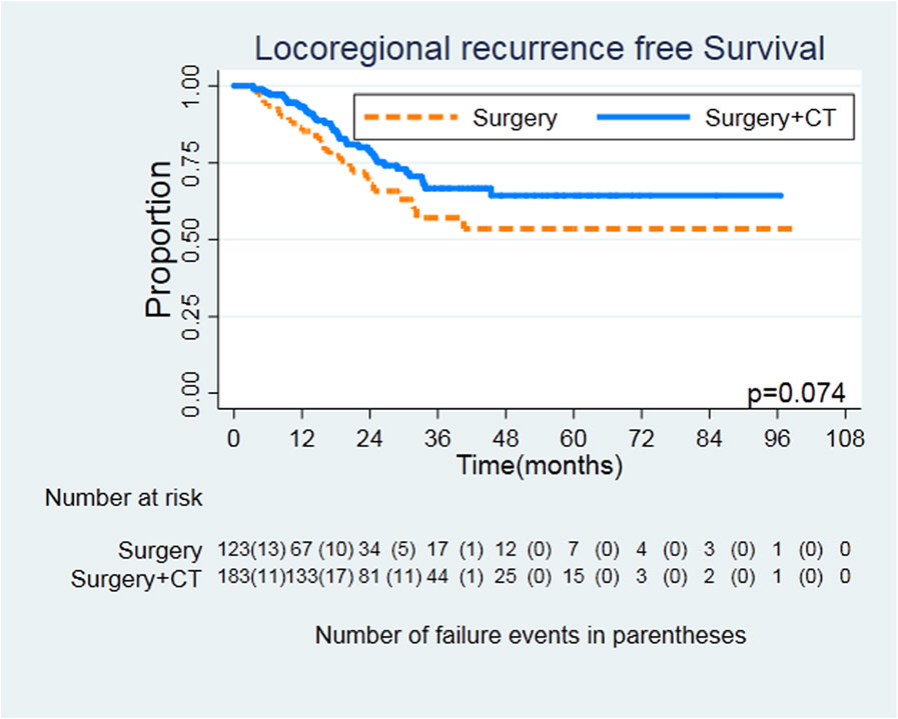
Locoregional recurrence-free survival for patients undergoing surgery or surgery + CT. Kaplan-Meier survival curves, HR = 0.648, 95% CI = 0.401–1.047, log rank *P* = 0.074

The median follow-up time was 17.1 months, and median RFS was 17.2 months (12.9 months in the surgery group and 19.6 months in the surgery plus chemotherapy group). Recurrence was found in 248 patients (81.0%), 107 (86.9%) in the surgery group and 141 (77.0%) in the surgery plus chemotherapy group, respectively. Within 730 days after surgery, the recurrence rate was 62.0% (190/306). The main recurrence pattern in both groups was distant metastasis. In the surgery group, 29 patients (23.5%) had local recurrence and 85 patients (69.1%) had distant metastasis. In the surgery plus chemotherapy group, 40 patients (21.8%) had local recurrence and 110 patients (60.1%) had distant metastasis. In both groups, seven and nine patients had local recurrence and distant metastasis at the same time. Figures 1 and 2 show the differences in RFS and distant metastasis-free survival (HR = 0.0.637, 95% CI = 0.495–0.820, *log rank P* = 0.0004; HR = 0.632, 95% CI = 0.476–0.839, *log rank P* = 0.0014). There was no difference in locoregional RFS between the two groups (HR = 0.648, 95% CI = 0.401–1.047, *log rank P* = 0.074) (Fig. 3).

### Site of tumor recurrence

Tumor recurrence was mainly through hematogenous dissemination. According to univariate Cox analysis, the incidence of hematogenous dissemination was 51.2% in the surgery group and 42.0% in the surgery plus chemotherapy group, with a significant difference between the two groups (HR = 0.519, 95% CI = 0.424–0.826, *P* = 0.002) (Table 2). There was no significant difference between the two groups for other recurrence sites: anastomosis, mediastinum, celiac axis, peritoneum, pleura, and nonregional lymph nodes, but the incidence in the surgery plus chemotherapy group was lower than in the surgery group.

**Table 2:** Results of univariate Cox regression analysis of RFS time per treatment arm

Site of recurrence	S (*n* = 123), *n* (%)	S + CT (*n* = 183), *n* (%)	HR	95% CI	*P* value
Anastomosis	10 (8.1)	14 (7.6)	0.664	0.294–1.497	0.323
Mediastinum	7 (5.6)	9 (4.9)	0.613	0.228–1.650	0.333
Celiac axis	12 (9.7)	17 (9.2)	0.615	0.290–1.305	0.205
Pleural	18 (14.6)	21 (11.4)	0.588	0.313–1.105	0.099
Peritoneal	5 (4.0)	7 (3.8)	0.712	0.226–2.249	0.563
Hematogenous	63 (51.2)	77 (42.0)	0.591	0.424–0.826	0.002
Nonregional lymph nodes	9 (7.3)	13 (7.1)	0.985	0.292–1.605	0.383

### Potential prognostic factors for RFS

Table 3 lists the potential factors affecting the recurrence of AEG patients after surgery. Univariate Cox analysis showed that treatment arm, age, pN stage, and pTNM stage were potential prognostic factors for recurrence. Multivariate Cox analysis using backward step regression analysis showed that pN stage (HR = 2.533, 95% CI = 1.951–3.289, *P* < 0.001) increased the risk of recurrence, while surgery plus chemotherapy (HR = 0.668, 95% CI = 0.519–0.861, *P* = 0.02) reduced the risk of recurrence. Therefore, according to the multivariate analysis, in stage III AEG patients, pN stage and surgery alone represented poor prognosis.

**Table 3:** Univariate and multivariable Cox regression analyses for RFS

Factor	Recurrence incidence (%)		Univariable		Multivariable	
	S	S+CT	HR (95% CI)	*P* value	HR (95% CI)	*P* value
Age, years (≦ 63/> 63)	80.7/91.5	74.1/81.6	1.451 (1.130–1.863)	0.03	1.282 (0.996–1.651)	0.054
Sex (male/female)	87.2/84.6	77.5/74.0	0.890 (0.610–1.298)	0.546	NA	
Histology (AC/mucinous AC)	85.7/92.0	76.0/80.0	1.072 (0.800–1.436)	0.642	NA	
Tumor length, cm (≦ 5.5/> 5.5)	88.5/84.9	76.8/77.2	0.979 (0.762–1.257)	0.865	NA	
pT stage (T2, T3/T4a)	92.9/73.6	78.5/75.0	0.804 (0.618–1.045)	0.103	NA	
pN stage (N0, N1, N2/N3)	82.4/93.8	64.3/98.5	2.624 (2.021–3.407)	< 0.001	2.533 (1.951–3.289)	< 0.001
pTNM stage (IIIa, IIIb/IIIc)	84.3/89.8	61.1/90.8	2.087 (1.614–2.698)	< 0.001	1.214 (0.804–1.833)	0.356
ALI (no/yes)	87.5/85.7	72.5/85.7	1.222 (0.938–1.592)	0.138	NA	
PNI (no/yes)	88.7/84.6	74.7/80.0	1.266 (0.984–1.627)	0.067	NA	
Method of resection (TTE/TAE)	88.5/84.9	88.2/66.1	0.799 (0.614–1.040)	0.096	NA	
Treatment arm (S/S+CT)	86.9	77.0	0.637 (0.495–0.820)	< 0.001	0.668 (0.519–0.861)	0.02
Number of resected lymph nodes (≦ 19/> 19)	87.1/77.0	86.8/77.1	0.954 (0.743–1.224)	0.712	NA	

## Discussion

Accurate preoperative staging of adenocarcinoma of the esophagogastric junction (AEG) is difficult, especially in lymph node staging. The postoperative treatment plan can be determined according to accurate pathological diagnosis in patients with high recurrence risk. Postoperative adjuvant chemotherapy aims to control potential micrometastasis after surgery and reduce the risk of local recurrence and distant metastasis.

We found that the main sites of local recurrence of AEG were anastomosis and mediastinum in the thoracic cavity (8.1% vs 7.6%, 5.6% vs 4.9%, in the surgery and surgery plus chemotherapy groups, respectively). The recurrence rate in lymph nodes around the celiac axis was 9.7% and 9.2% in the surgery and surgery plus chemotherapy groups, respectively, suggesting that Siewert II AEG may metastasize both downward and upward, showing bidirectional features. Multicenter studies [13] have shown that proximal gastrectomy, lower esophageal resection, and local lymph node resection are the minimum requirements for surgical treatment of AEG. Expanding esophagectomy and enlarging the scope of lymph node dissection do not improve local recurrence [14]. Other studies [15] have shown that the overall survival is not significantly related to the dissected mediastinal lymph nodes. In our study, there was no difference in the recurrence rate between patients treated with transthoracic or abdominal surgery. However, the local recurrence rate in the surgery group was higher than that in the surgery plus chemotherapy group (23.5% vs 21.8%). Although the Kaplan-Meier curve showed no significant difference in local recurrence between the two groups, the survival curve of the surgery plus chemotherapy group was above that of the surgery group, suggesting that the surgery plus chemotherapy had a tendency to reduce local recurrence, and the effect was not significant, which might be related to the surgical injury and blockage of blood vessels and lymph nodes in the surgical area, which to some extent affect inhibition of potential micrometastasis in the surgical area by chemotherapeutic drugs. Currently, neoadjuvant chemotherapy and neoadjuvant radiochemotherapy are the hotspots in the field of cancer research. There is also evidence [16–18] to indicate that neoadjuvant chemotherapy or neoadjuvant radiochemotherapy can reduce local recurrence of AEG and improve prognosis, but there is still controversy about this treatment [19]. Due to the fear of cancer [20] and concern about complications associated with neoadjuvant therapy, neoadjuvant chemotherapy in our medical center is not smoothly administered. Only 2.4% of our patients received neoadjuvant chemotherapy; therefore, adjuvant treatment after surgery is still valuable in patients.

Patients with AEG have a high risk of hematogenous dissemination after surgery. Liver metastasis can occur as early as 1 month postoperatively. Distant metastasis sites vary, mainly the liver and lungs, with multiple subcutaneous, ovarian, and small intestinal metastases being less common. Compared with surgery alone, surgery plus chemotherapy reduced hematogenous dissemination (51.2% vs 42.0%). Distant pleural and peritoneal metastases occurred in some patients via implantation or hematogenous dissemination of cancer cells. But whether implantation of cancer cells occurred during surgery or was caused by long-term hematogenous dissemination is unclear. In univariate Cox analysis, it was found that the ratio of pleural and peritoneal metastases in the surgery plus chemotherapy group was lower than that in the surgery group (3.8% vs 4.0%, 11.4% vs 14.6%) but these differences were not significant (*P* = 0.563, 0.099).

In our study, within 24 months after surgery, most patients had a recurrence (62.0%, 190/306): 72.3% (89/123) in the surgery group and 55.1% (101/183) in the surgery plus chemotherapy group, indicating that advanced AEG has high potential of early recurrence. Previous studies have shown that survival rates of patients with tumor recurrence are low, especially in those with earlier recurrence [21, 22]. Multivariate Cox analysis showed that patients with higher pN stage were more prone to recurrence. Surgery plus chemotherapy may be beneficial to RFS, but the recurrence rate was still high. Therefore, for AEG patients, we should focus on pN staging, modify the follow-up protocol within 2 years after surgery, and increase the frequency of follow-up appropriately, so as to find timely evidence of recurrence and formulate subsequent treatment strategies. At the same time, we found an interesting phenomenon in that the age of patients in the surgery plus chemotherapy group was lower than that in the surgery group (median age 62 years vs 65 years, *P* < 0.001). Univariate Cox analysis showed that older age was associated with recurrence after surgery. This indicated that the younger patients had higher compliance with subsequent treatment, thus improving the prognosis.

This retrospective study had some limitations: although there were inclusion criteria, there may have been selection bias; for example, patients who were lost to follow up may have had no recurrence. Unfortunately, we were unable to obtain sufficient data on follow-up treatment after relapse. This study was conducted in a single center, which helped us to understand the recurrence mode of advanced AEG and improve subsequent treatment strategies. Strengthening health education and improving patient compliance, and increasing the surgical area (including lower mediastinum and upper abdomen) for radiotherapy may also help to reduce local recurrence. However, the ARTIST (Adjuvant Chemoradiation Therapy in Stomach Cancer) trial [23] found that additional postoperative radiotherapy had the same benefit as chemotherapy alone in preventing recurrence of gastric cancer. Therefore, while focusing on randomized clinical trials [24, 25], we also need to develop and optimize treatment strategies that accord with the medical conditions of the local area. We found that stage III AEG mainly metastasizes to distant sites, predominantly through hematogenous transmission. We recommend increasing the number of follow-up visits within 2 years after surgery, especially in the second year. Surgery plus chemotherapy can improve RFS and reduce distant metastasis, but they do not have a beneficial role in controlling local recurrence.

## Conclusions

Patients with AEG have a high risk of hematogenous dissemination after surgery. Postoperative treatment arm and pN stage were independent risk factors in patients with AEG. Surgery plus chemotherapy may be beneficial to RFS, but the recurrence rate was still high. For AEG patients, we should focus on pN staging, and the future direction in this field will probably focus on modifying the follow-up protocol after surgery, strengthening health education and improving patient compliance, and increasing the surgical area for radiotherapy.

## Data Availability

The data used and analyzed in the current study are available from the corresponding author upon reasonable request.

## Abbreviations

AEG: Adenocarcinoma of the esophagogastric junction
RFS: Recurrence-free survival
S: Surgery
CT: Chemotherapy
HR: Hazard ratio
CI: Confidence interval
AC: Adenocarcinoma
p: Pathologic
c: Clinical
ALI: Angiolymphatic invasion
PNI: Perineural invasion
TTE: Transthoracic esophagoastrectomy
*n*: Number
TAE: Transabdominal esophagoastrectomy
NA: Not applicable
